# Incomplete pneumolysin oligomers form membrane pores

**DOI:** 10.1098/rsob.140044

**Published:** 2014-04-23

**Authors:** Andreas F.-P. Sonnen, Jürgen M. Plitzko, Robert J. C. Gilbert

**Affiliations:** 1 Division of Structural Biology, Wellcome Trust Centre for Human Genetics, University of Oxford, Roosevelt Drive, Oxford OX3 7BN, UK; 2 Department of Molecular Structural Biology, Max Planck Institute of Biochemistry, Am Klopferspitz 18, Martinsried 82152, Germany; 3 Centre of Chronic Immunodeficiency, University of Freiburg, Breisacher Strasse 117, Freiburg 79106, Germany; 4 Bijvoet Center for Biomolecular Research, Utrecht University, Padualaan 8, 3584 CH Utrecht, The Netherlands

**Keywords:** pneumolysin, pore formation, cryo-electron tomography, proteolipid pore, toroidal pore

## Abstract

Pneumolysin is a member of the cholesterol-dependent cytolysin (CDC) family of pore-forming proteins that are produced as water-soluble monomers or dimers, bind to target membranes and oligomerize into large ring-shaped assemblies comprising approximately 40 subunits and approximately 30 nm across. This pre-pore assembly then refolds to punch a large hole in the lipid bilayer. However, in addition to forming large pores, pneumolysin and other CDCs form smaller lesions characterized by low electrical conductance. Owing to the observation of arc-like (rather than full-ring) oligomers by electron microscopy, it has been hypothesized that smaller oligomers explain smaller functional pores. To investigate whether this is the case, we performed cryo-electron tomography of pneumolysin oligomers on model lipid membranes. We then used sub-tomogram classification and averaging to determine representative membrane-bound low-resolution structures and identified pre-pores versus pores by the presence of membrane within the oligomeric curve. We found pre-pore and pore forms of both complete (ring) and incomplete (arc) oligomers and conclude that arc-shaped oligomeric assemblies of pneumolysin can form pores. As the CDCs are evolutionarily related to the membrane attack complex/perforin family of proteins, which also form variably sized pores, our findings are of relevance to that class of proteins as well.

## 2. Introduction

Pore formation is a strategy for host infection and the instigation of disease employed by many pathogenic organisms, and especially by bacteria [1,2]. One of the largest and most intensely studied families of pore-forming proteins are the cholesterol-dependent cytolysins (CDCs) first identified in the Gram-positive bacterial genera *Streptococcus*, *Clostridium*, *Listeria* and *Bacillus* [3,4]. Prominent examples of CDCs include listeriolysin from *Listeria monocytogenes* and perfringolysin from *Clostridium perfringens*, as well as pneumolysin from *Streptococcus pneumoniae*. In each case, the toxin is a pathogenicity determinant of the producing bacterium, with its toxic effect being tightly correlated with the capacity to induce pores in target membranes [5–7]. The CDCs are also highly immunogenic and are vaccine candidates for their producing organisms [8], while listeriolysin in particular is being employed in vaccines against other diseases, including tuberculosis and cancer, primarily because of its capacity to confer intracellular growth [9–11].

Work over many years has shown that the CDCs form pores of variable size. Although, as discussed later, there is a well-defined mechanism for the formation of pores approximately 30 nm in diameter via a ring-form oligomeric assembly, the ability of the same proteins to generate much smaller pores, of variable size from approximately 1–2 nm across upwards, remains unexplained. Among the evidence for the formation of both small and large pores by CDCs are single-channel conductance studies which show different-sized conductance step-changes within the same experiment *in vitro* [12–16] and on cells [17], and studies in cells in which both small and large pores have been observed during infection with *Listeria* [5]. As previously observed [15,18,19], the capacity of the same protein to form pores of variable size could be explained by pore formation using both the full-ring and the arc (incomplete ring) oligomers observed by electron microscopy [3,20,21]. This paper describes a study designed to test whether this is the case by imaging pores of the CDC pneumolysin *in situ* in membranes, without stain or model bias derived from iterative two-dimensional image alignment [22].

**Figure 1.**
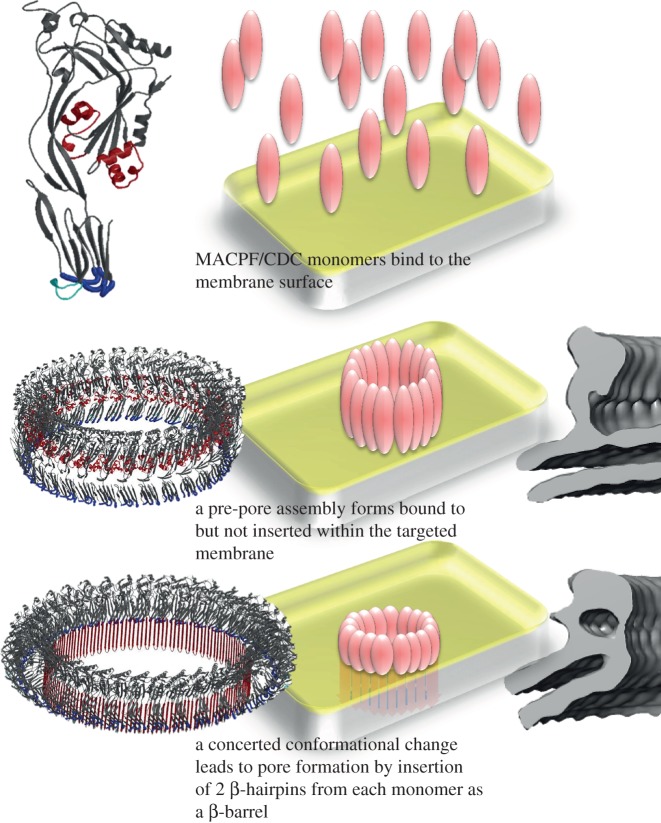
Schematic of pore formation by CDCs (and MACPF proteins). In the left-hand column, we show the atomic structure of perfringolysin [23] (top) with the helices forming transmembrane hairpins on pore formation coloured red and the regions involved in membrane binding cyan and blue; and a model of the pre-pore (middle) and pore (bottom) with the transmembrane β-hairpins deployed. In the central column, we show schematic diagrams for monomers binding to the membrane (top), forming a pre-pore (middle) and a pore (bottom). To the right, we show in grey the subunit profiles of the pre-pore and pore states, which are distinctively different. The pre-pore has a comma-like profile very similar to the atomic model of perfringolysin, for example [23], while the pore has a more compact and thinner profile.

The mechanism of pore formation for CDCs has been defined to date with respect to the larger pores formed by full rings of oligomerized subunits (figure 1). Their conversion from a water-soluble to a membrane-inserted form requires the refolding of a set of α-helices into a pair of transmembrane β-hairpins (TMHs), creating a continuous β-sheeted wall to the pore [24,25]. This process occurs only once the formation of a pre-pore oligomeric ring is completed [26,27] and involves a doubling-over of the CDC subunit in order to bring the TMHs to an appropriate position to span the membrane bilayer [28,29]. Is this mechanism of pore formation able to accommodate a role for incomplete rings? As previously discussed [3,18,30], it can because all that need differ between a full-ring and an arc forming a pore through a membrane is the point at which membrane insertion occurs, and this is probably governed by a kinetic mechanism and relates to the CDC concentration and membrane fluidity.

In the work described here, we derived low-resolution maps of pneumolysin oligomers on the surfaces of liposomes by sub-tomogram averaging. This procedure allowed an objective estimation of the status of the membrane within the curve of the membrane-bound oligomers. Thus, pre-pores could be distinguished from pores by the presence of sealing membrane across the inside of the oligomer, and by these means we show that pores are fostered by incomplete oligomers, arcs, as well as by complete rings of subunits. Therefore, the capacity of CDCs (and proteins to which they are related such as the membrane attack complex/perforins (MACPFs)) to form pores of variable size could be explained by pore-forming oligomeric arcs that generate a proteolipid structure [3,18].

## 3. Results

### 3.1. Tomogram generation and sub-volume identification

**Figure 2.**
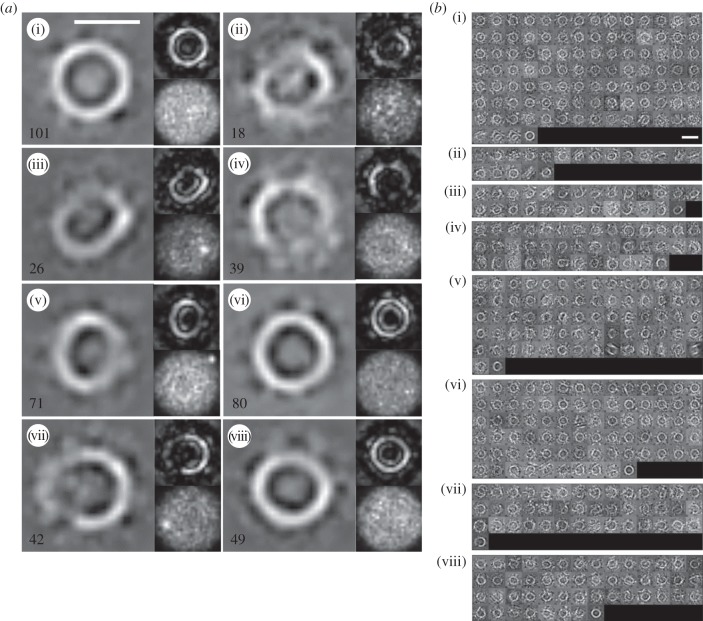
Classification of two-dimensional projections of sub-tomogram averages. (*a*) The main images show class averages of the projections and display complete rings as well as arcs (incomplete rings) of protein. The insets in each case are the corresponding statistical I-image, calculated using IMAGIC software [33] and as defined in the Material and methods (above), and the class variance (below). The number of projections found in each class is also given on each average and the bar in the first image indicates 250 Å. (*b*) The members of each of the classes shown in panel (*a*); the class average is shown again as the final image in each montage, and the bar in the first montage indicates 500 Å.

Purified pneumolysin was added to cholesterol-containing liposomes and incubated at 37°C for 5 s or 1 min prior to the collection of cryo-electron tomograms (see Material and methods for a full description of data acquisition and analysis). Longer incubation times result in sample aggregation; this phenomenon has not yet been explained but has been observed for pneumolysin using both spectroscopic and imaging methods and relates to a tendency of targeted membranes to bleb [28,31,32]. Following reconstruction (see the electronic supplementary material, Movies S1 and S2 for examples), we interactively selected from 34 different tomograms 1953 sub-volumes containing oligomers, taking care to choose examples seen both in profile and with the oligomeric ring viewed from above in the frame of reference. Projection images of top-view sub-volumes, when classified using IMAGIC software (see Material and methods) [33], clearly demonstrated the presence of rings and arcs of protein density both in the class averages and the statistical I-images, while the variances of each image showed no significant signal (see Material and methods) (figure 2*a*). Furthermore, the individual images from each class match well with their corresponding class average (figure 2*b*). This exercise suggested that both complete and incomplete oligomers were present in the sample and could be resolved from one another, and we therefore decided to perform three-dimensional volume classification and averaging, making use of a recently developed maximum-likelihood methodology [34].

### 3.2. Sub-volume classification

**Figure 3.**
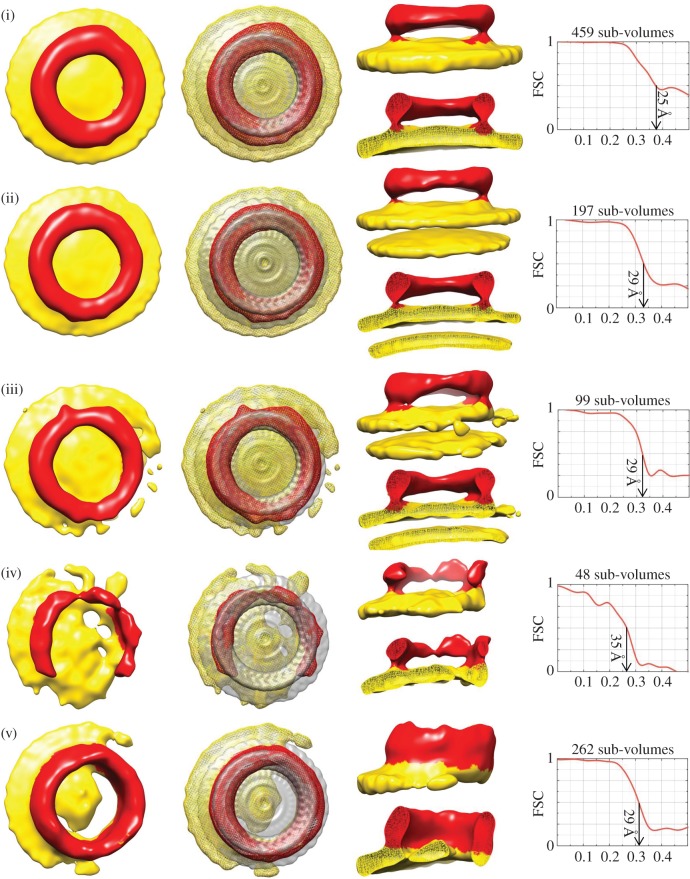
Sub-tomogram class averages of pre-pore and partial pore structures observed *in situ* on liposomal membranes. Five different sub-classes representing pre-pores or partial pores, comprising in total 1065 sub-tomogram volumes, were identified in the whole dataset of 1953. They are shown viewed from above alone (first image from left) and with the pre-pore single-particle reconstruction [28] superimposed (second image), and from the side both as a whole and sectioned through the middle (upper and lower views in the third panel of each row). Protein is coloured red and membrane yellow. Maps varied in the completeness of the pre-pore oligomer (iv), the presence of a second membrane bilayer as in multilamellar liposomes ((ii) and (iii)) and the extent to which the membrane remained intact ((v) appears to be a transitioning partial pore, and (iv) may be also). The numbers of sub-volumes in each class map and their resolution together with the Fourier shell correlation (FSC) for each case are shown on the right-hand side.

**Figure 4.**
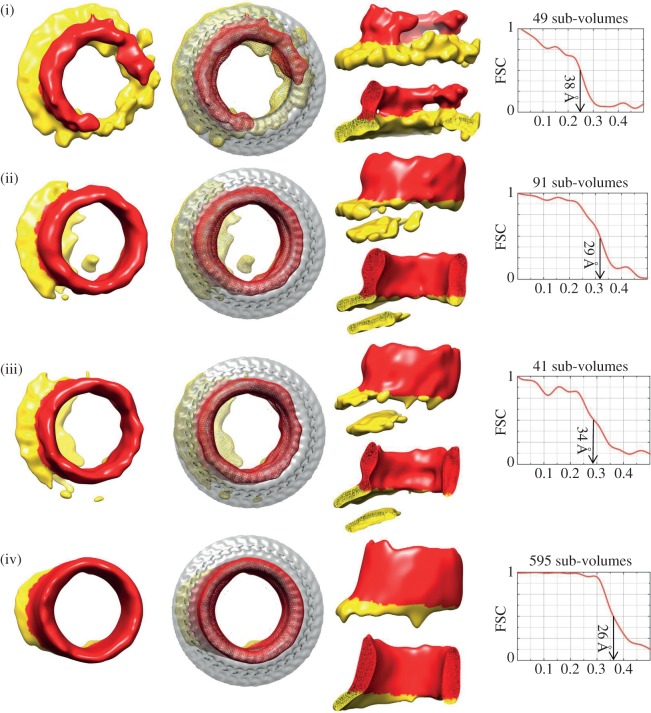
Sub-tomogram class averages of pore structures observed *in situ* on liposomal membranes. Definition as a pore was based on the absence of a membrane within the curve and on the plane of the oligomeric arc or ring. Pores are shown viewed from above alone (first image from left) and with the pore single-particle reconstruction [28] superimposed (second image), and from the side both as a whole and sectioned through the middle (upper and lower views in the third panel of each row). Protein is coloured red and membrane yellow. The numbers of sub-volumes in each class map and their resolution together with the Fourier shell correlation (FSC) for each case are shown on the right-hand side.

We subjected the whole dataset to iterative three-dimensional classification and alignment with the software XMipp (see Material and methods) [34]. We started with alignment to an average of all the sub-volumes and, taking care to keep the oligomers centred, ultimately identified 12 distinct class averages; in the paper, we show nine class maps from 1841 sub-volumes or 94% of the data (figures 3 and 4). The sub-tomographic class average maps were characterized by a planar structure from which ring- or arc-like density projected; the former was obviously identifiable as the bilayer surface, the latter as the pneumolysin oligomer. Some of the maps (figure 3, volumes (ii) and (iii) and figure 4, volumes (ii) and (iii)) had two lipid surfaces because they were associated with double-membraned vesicles (see the electronic supplementary material, Movies S1 and S2 also). Furthermore, while some of the pneumolysin oligomers were in a pre-pore state, others were in a pore-forming state, as indicated by the presence or absence of a membrane plugging the centre of the oligomeric ring [28]. However, as in the two-dimensional analysis (figure 2), the morphology of the pneumolysin oligomer varied between a partial ring (an arc) of protein and a full ring. We assessed the resolution of each sub-tomographic volume by Fourier shell correlation (figures 3 and 4) and they varied between 25 and 38 Å.

### 3.3. Pre-pores and partial pores

A total of 1065 sub-volumes were classified as having a membrane either completely (figure 3(i)–(iii)) or partially (figure 3(iv) and (v)) extended across the oligomeric arc or ring. Because the membrane extends right across the inside of their oligomeric curve, structures (i)–(iii) are identifiable as pre-pores. By contrast, because the membrane extends only partly across the oligomer, structure (v) is a partial pore (a pore that has started to open) and structure (iv) may be as well. One thousand and four of the pre-pore/partial pore sub-volumes averaged to generate maps with more-or-less complete oligomers (94% of the total; averages (i)–(iii) and (v)) while 61 (6%) were classified as incomplete and gave a class average shaped like a letter ‘C’ (e.g. average (iv); another similar map is not shown that resulted from 13 sub-volumes—pre-pore (vi) (see data refinement scheme in the electronic supplementary material).

We resolve clearly the membrane skirting the base of the pneumolysin oligomer in each case. This, combined with the resolution of secondary membranes in the structures bound to the outside of double-membraned vesicles, clearly shows that we can discriminate between the protein arc/ring and the lipid bilayer(s) present in the class averages. In turn, this implies that the sub-classification has identified 55% (1078/1953) of the sub-volumes as belonging to a category of pre-pores and partial pores.

### 3.4. Pores

In contrast to the pre-pores and partial pores, a total of 875 volumes were classified to yield average structures in which the inner curve of the pneumolysin oligomer provided a perimeter for a membrane pore—an open hole through the membrane beneath it (figure 4). This equates to 45% of the classified volumes, which essentially agrees with the proportion of pores over pre-pores (43%) found in a previously published single-particle reconstruction study of pneumolysin oligomers on liposomes [28]. The membrane-bound pneumolysin oligomers in that study were generated by the same protocols as those used in the current work [28]. We have again numbered the pore class sub-volumes identified (numbers (i)–(iv) are shown in figure 4 of a total of six). Of these, that numbered (i) was substantially incomplete and had a significant ‘skirt’ of lipid bilayer all around the pore-forming oligomer so that the lipid closing the pore edge on the far side of the protein arc was also resolved. The volume shown in figure 4(i) derives from 49 sub-volumes; in addition, another 99 sub-volumes averaged into two classes displaying arc morphology with no central lipid bilayer and therefore containing a pore (pores (v) and (vi)). However these are not shown because the lipid completing the pore on the side opposite the protein arc was in these cases averaged out (we discuss below why the lipid regions of the pore structures are less well resolved than the protein regions). In total, therefore, pore-forming protein arcs account for 148 sub-tomogram volumes or 17% of the pores. Averages (ii)–(iv) were more-or-less complete oligomers from 727 volumes. Pore averages (ii) and (iii) in figure 4 have, like pre-pores (ii) and (iii), a second membrane showing that they are inserted into multilamellar vesicles. Despite multiple attempts, we could not sub-classify the class map shown in figure 4(iv) any further.

Because we, again, resolve membrane regions among the pore classes skirting the oligomers but clearly do not observe membrane sealing the inside of the pneumolysin oligomer, we conclude that this sub-classification identifies pore structures. However, a clear trend seen in the partial pore and pore structures is that relatively less density is resolved skirting these structures than in the pre-pores that have full coverage of the inside of the oligomer by sealing lipid, and better membrane density is observed on one side of the oligomer rather than the other in several cases. But, clearly, these structures are not free of membranes because they have been directly picked from the surfaces of liposomes (electronic supplementary material, Movies S1 and S2). What could explain this trend? Clearly, in pores a greater proportion of the visualized density is from protein rather than lipid (because the lipid has disappeared from the inside of the oligomeric ring during pore formation) and this seems to be dominating the alignment, resulting in the averaging-out of the (more fluid, less structured) remaining lipid density. This is clearest for the class of 595 pore-forming rings without a second membrane in figure 4(iv). However, a significant proportion of the pore structure is lipid rather than protein when arcs form pores and this has enabled us clearly to resolve lipid completing an arc pore on the far side from the protein density (figure 4(i)), which confirms the genuine pore-forming capacity of oligomeric arcs.

### 3.5. Membrane-bound pneumolysin oligomer tomograms compared to single-particle reconstructions

The subunit profiles in the pre-pore and pore states of pneumolysin oligomers are distinctively different, as shown in figure 1. The pre-pore oligomers in figure 3(i)–(iii) show similar comma-shaped profiles to those resolved in the previously determined single-particle reconstruction, with a comma-like shape [28]. By contrast, the pore oligomers (figure 4(i)–(iv)) show a more linear profile that reflects the deployment of the TMHs to form a (partial or complete) β-barrel as also seen in the single-particle reconstruction (figure 1) [3,28]. Interestingly, the oligomers that are partially pore forming (figure 3(iv) and (v)) show a pre-pore, comma-shaped subunit profile on the side where bilayer still extends from the concave surface of the oligomer, but a more sheer, pore-like profile on the side where lipid seems to have retreated from the inserted protein, in agreement with a partially pre-pore and partially pore-forming state.

## 4. Discussion

In this paper, we describe an electron tomography analysis of the structures of pneumolysin oligomers bound to model lipid membranes. By sub-tomogram classification and averaging, we show that pre-pore and partial pore structures, on the one hand, can be distinguished from pores, on the other. This distinction is based on the presence of membrane within the circle of the protein oligomer in pre-pores and partial pores, and no internal membrane in the pores although it is still resolved skirting the outside of pore-forming oligomers. We have furthermore shown that pre-pore-forming and pore-forming oligomers are observed as both incomplete oligomeric arcs and complete oligomeric rings. These data strongly support the contention that membrane-bound oligomeric arcs of CDCs such as pneumolysin are capable of forming pores [3,18,30]. They also support the argument that such structures explain the capacity of CDCs and related proteins to form small- as well as large-conductance pores [12–16].

The identification of pre-pore and pore stages to CDC activity, using perfringolysin, first suggested that their pore formation is an all-or-nothing (quantized) event [26,29]. One of the major objections to the idea that arciform oligomers of CDC subunits can generate pore structures has been that this would not fit with stepwise pore formation; however, as discussed above and elsewhere, if a pre-pore can as well be an arc oligomer as a ring oligomer then this objection is lifted [3,18,30]. Another objection is that the structures formed would be unstable. Leaving aside the fact that this may not be a problem for the biological function of the pore (short-lived lesions in membranes could easily have long-term effects, as in the activity of perforin itself during cytotoxic T-cell mediated apoptosis [32,35,36]), this assertion is not supported by the available data. It is clear from multiple published studies that CDCs like pneumolysin and listeriolysin form pores of variable functional diameter and therefore electrical conductance that are stable enough to have their conductance and functional properties measured [5,14,15,17]. Yet, the ring-form oligomers are sufficiently consistent in their sizes that they cannot explain the widely variable bore of individual functional pores [28]. On the other hand, arc oligomers provide a potential mechanism whereby pores of differing sizes could be generated by a single protein. Thus, although it could be objected that the physiological relevance of pore-forming arciform oligomers is unknown, in fact the physiological relevance of rings forming pores is just as unclear. All that *is* known is that the CDCs generate pores in membranes, not what form they have. The already-cited evidence for the capacity to form pores of varying size is good evidence that arcs (which have this capacity) are highly relevant physiologically.

**Figure 5.**
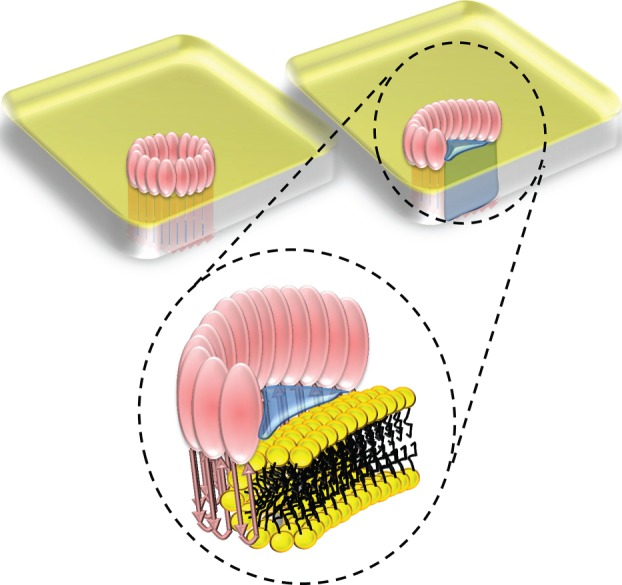
Implications for pore formation by CDCs. Schematic of a pore formed by a ring of pneumolysin subunits (top left), by an arc of subunits with a toroidal lipid edge (top right) and a close-up of the toroidal structure, as also found in pores formed by electroporation and by proteins such as Bax, equinatoxin II and colicin E1 [3,37–42] and truncated *α*-haemolysin [43].

Data on a variety of other pore structures indicate that proteolipid pores—consisting of matrices of protein and lipid—do exist and are characterized by a toroidal arrangement of the lipids themselves, as shown in figure 5. This solution to pore formation by CDCs was first proposed in 1985 by Bhakdi *et al*. [20] and supported by their later work which showed how truncated (arc) oligomers of streptolysin form functional pores of reduced size [19]. Experimental evidence that such a lipid arrangement is possible now comes from a variety of sources, including X-ray diffraction studies of the α5 helical peptide derived from pro-apoptotic Bax [37], viscoelastic studies of membranes with the bee-venom peptide melittin [44], transbilayer lipid dynamics in the presence of the *Xenopus* antimicrobial peptide magainin [45], NMR and FTIR studies of the sea anemone protein equinatoxin II [38] and the effect that lipids promoting toroidal lipid structures have on colicin E1 pore formation [39]. The toroidal form of lipid structure is also expected during electroporation [40] and to exist during membrane fusion [46], which suggests that it can persist for sufficient lengths of time to play a significant role in CDC activity. A recent single-particle reconstruction of the proteolipid pores formed by full-length Bax [41] and imaging analysis of Bax pores formed in giant unilamellar vesicles over periods of hours [42] further demonstrate that the lifetime of such structures is sufficient for a biologically relevant effect. Both α-helix-based (as in Bax) and β-sheet-based (as in CDCs) mechanisms of pore formation appear capable of proteolipid pore formation; a recent study showed this even for the canonical β-barrel pore-forming protein α-haemolysin [43] while another recent report described simulations using β-sheet arcs of protegrin which supported the formation of pores via a toroidal lipidic-structure-based mechanism [47].

Stabilization of a protein/torodial lipid composite pore will be critically dependent on the interactions between the open ends of the protein arc and the hydrophobic interior of the lipid bilayer. This may be enabled by a similar arrangement to the one we recently reported for the pore-forming protein lysenin interacting with the acyl chain of sphingomyelin in which the chain was ring-stacked onto a pair of tyrosines [48]. In pneumolysin, the first TMH has three aromatic residues (F165, F169 and F180) and TMH2 contains two (F263 and W278); other MACPF/CDCs have up to eight aromatic residues in their equivalent regions [3] and by a similar mechanism to that observed in lysenin these could play a key role in stabilizing the protein : lipid interface.

The observation of forms of pneumolysin oligomeric ring that are apparently partially inserted to form a pore (24% of the total pre-pores at least; figure 3 image (v)) is very interesting as it suggests a possible mechanism for the removal of lipids from the interior of the oligomeric pore-forming ring. Clearly, when arcs insert, the lipid can retreat from the hydrophilic TMH surface to leave a pore, like oil from water, but the fate of lipids when a ring of subunits inserts has never been explained [4] though it is clear that lipids undergo dramatic rearrangements when CDCs act [31,49]. If the CDCs insert into membranes in a rolling fashion, as is also proposed for the related membrane attack complex [50], then this would provide an explanation: rather than hole-punching lipids from the oligomer interior the lipids would be able to flow into the surrounding membrane as the oligomer settled into a transbilayer orientation. This possibility will require further investigation but is not prevented by data showing that oligomers form pores in an all-or-nothing fashion, because studies performed to date have not had the time resolution to capture the retreat of lipids from under the inserting oligomer into the rest of the membrane [26,27].

The significance of our findings is not limited to the CDCs, because of their relationship to the MACPF family of proteins; the CDC/MACPF superfamily seems to have remained conserved in structure since the last common ancestor of eubacteria and humans, at least [3], and the conservation appears to encompass both the pore-forming and membrane-binding domains of the proteins [51]. For the MACPF protein perforin, too, pores of variable functional size have been noted in biophysical and cell biology studies [32,36,52] and arciform oligomers are also observed for both the MAC itself and perforin [53–55]. Not only, therefore, can oligomeric arcs provide an explanation for the variable functional pore sizes observed for CDCs like pneumolysin and listeriolysin *in vitro* [13–15] and in cells [5,17], but also for the variable pore sizes seen in the related perforin using also biophysical [32,36] and cell biological [35,52] approaches.

## 5. Material and methods

### 5.1. Sample preparation

Pneumolysin was expressed and purified as previously described [56]. Pneumolysin was incubated with single unilamellar vesicles (SUVs) containing egg phosphatidylcholine : cholesterol : dicetyl phosphate in molecular ratios of 10 : 10 : 1 at a cholesterol to protein ratio of 100 : 1. Note however that, as shown by the tomograms determined below (see the electronic supplementary material, Movies S1 and S2), some of the SUVs were double layered. SUVs were prepared as follows: a fine lipid film was formed under a stream of argon, dried in a desiccator, solubilized with sterile phosphate buffered saline, and subjected to 10 iterative freeze–thaw cycles followed by repetitive extrusion (at least 11 passes) at 37°C through 100 nm polycarbonate filters (Avanti Polar lipids). Protein/liposome incubations were performed at 37°C for 1 min or 5 s (directly on the EM grid in a humidity chamber) as reported previously [28]. Longer timeframes result in sample aggregation [28,31]. Prior to application of the proteoliposomal suspension and vitrification in liquid ethane by plunge freezing, lacey carbon or C-Flat copper grids (Plano, Wetzlar, Germany) were loaded with a suspension containing 10 nm colloidal gold clusters.

### 5.2. Data capture and tomogram generation

Cryo-tomographic tilt series were acquired on a Tecnai Polara transmission electron microscope (FEI Company Inc., Hillsboro, OR, USA) operated at 300 keV, equipped with a post-column GIF2002 energy filter and a slow scan 2048 × 2048 pixel Multiscan CCD camera (Gatan Inc., Pleasanton, CA, USA). Images were recorded in zero-loss mode (energy filter slit width: 20 eV) under low-dose conditions at a final magnification of 64 171, resulting in a pixel size of 4.72 Å on the object level. Tilt series taken at an intended underfocus of −8 μm comprised projections from −65° to 65° with an angular increment of 1.5°. For data acquisition, the Xplore3D software (FEI Company Inc.) was used. Projection alignment using at least eight gold markers and tomographic reconstruction by weighted back projection was done using the procedures implemented in the TOM Toolbox [57].

### 5.3. Sub-tomogram volumes selection and classification

Tomograms were displayed using the Bsoft program Bshow [58]. Individual pores were picked manually to include both top views of the ring and side views of the profile of the oligomers.

Classification of projection images of the sub-volumes in two dimensions was performed in IMAGIC [33] using standard multivariate statistical analysis protocols. To make an assessment of the class average quality, I-images and variances were computed. Whereas in ‘S images’, high densities correspond to the most significant image areas (‘the most stable areas throughout the set of summed images’) and are calculated as
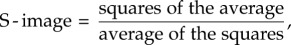
an I-image gives the amount of information collected in the different parts of the images in bits:
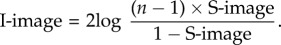


Both the I-images and variances (figure 2*a*) and the appearance of the individual two-dimensional images analysed which formed the classes shown in figure 2*b* validate the actual existence of arc views of pneumolysin oligomers.

The Xmipp package was used for three-dimensional data analysis [34]. In preparation, missing wedges were determined for each tomogram using the program xmipp_detect_missing_wedge. The missing wedges were then applied to each sub-volume depending on its tomogram of origin throughout the classification procedure. Iterative rounds of classification made use, first, of interpolated images with pixel size 18.72 Å and latterly of full-resolution images with 4.72 Å per pixel. We employed both a reference-free start with a set of 197 top-view sub-volumes (automated in Xmipp and using an average of all sub-volumes included) and latterly a referenced alignment using the pneumolysin single-particle pre-pore and pore reconstructions [28] deposited in the EBI macromolecular structure database (http://www.ebi.ac.uk/pdbe/emdb/) with codes EMD-1106 and EMD-1107. The two strategies gave very similar results, but the *ab initio* approach resulted in slightly better resolution maps and those are the structures depicted in this paper. For comparison, however, the sub-tomogram average maps have been superposed with the single-particle maps in figures 3 and 4, from which they are otherwise wholly independent.

Each round of alignment either *ab initio* or with a set of initial assigned parameters was performed using the following settings, derived from the program documentation: five iterations, an angular increment of 15°, an initial regularization parameter of 5 N/K^2^ and final regularization parameter of 0 N/K^2^ with five regularization steps. Note that, importantly, in the initial alignments with finite numbers of references less product classes resulted than references used. Resulting maps were inspected visually and if they consisted of large numbers of particles then further sub-classification without alignment was performed. The number of sub-classes imposed was determined empirically—if the refinement terminated after between one and three iterations then the number was reduced or increased until proper convergence could be achieved (after 14–98 iterations); up to 200 iterations were allowed for these cycles of sub-classification. For the sub-classifications without alignment, the same parameters were used as for the actual alignments but with the tags –keep_angles and –don't_align. The electronic supplementary material gives a dendrogram of the final three major cycles of alignment. Thus, alignment of 1953 sub-volumes with five references gave three populated sub-classes (with 185, 955 and 813 members). These were sub-classified so that the 153 volumes gave three classes (with 74, 39 and 72 members), the 955 volumes six classes (with 491, 60, 55, 158, 110 and 81 members) and the 813 volumes three classes (with 135, 545 and 134 members). Because the sub-classes derived from 813 volumes all resembled others represented by sub-classes of the 185 and 955 volumes, only those nine sub-class averages were taken forward as alignment references.

The whole dataset was then aligned again with nine reference volumes, at the asterisk in the dendogram. This again resulted in three populated classes. These were sub-classified as follows: one (pore) group contained 54 members and appeared the same as another pore structure with 307 members so was not used in the final alignment. The second group contained 1123 members and was sub-classified into eight classes, all of which were used in the final alignment. The third contained 776 members and was initially sub-classified into five classes of which one had 501 members and could be further subdivided into five further classes with 93, 27, 93, 31 and 307 members. Seventeen maps were then taken forward for a final round of alignment and classification, as shaded in the data refinement scheme in the electronic supplementary material, with co-centring of the oligomeric arc or ring and the centre of the sub-volume box being reconstructed. This stage in the analysis is marked by a double asterisk.

Four populated classes resulted this time from sub-classification of the aligned data. One, consisting of 1028 images, could be broken down into six sub-groupings, five pre-pore states and one pore state. The second, consisting of 253 images, could be separated into three pore and one pre-pore state; a third state consisted of an (arc-formed) pore and 77 images and the final (595-member) class could not be further sub-classified and showed a ring-oligomer pore. Pores (v) and (vi) consisted of arc-shaped oligomers but because the lipid on the far side of the pore from the protein arc was averaged out in them they are not shown in the paper; pre-pore (vi) was another arc oligomer but is not shown in the paper as it consisted of only 13 images. The resolution of all the maps was determined using Fourier shell correlation and the corresponding plots are shown in figures 3 and 4.

Figures were prepared using UCSF Chimera software [59].

## Supplementary Material

Data refinement scheme

## Data accessibility

The reconstructions described in this paper are deposited in the Electron Microscopy Data Bank, http://www.ebi.ac.uk/pdbe/emdb/, with deposition codes EMD-2611, EMD-2612, EMD-2613, EMD-2614 and EMD-2615 (pre-pores) and EMD-2616, EMD-2617, EMD-2618 and EMD-2619 (pores).
