# Supplementary material for: Incomplete pneumolysin oligomers form membrane pores
Source: Open Biol. 2014 Apr 23;4(4):140044. doi: 10.1098/rsob.140044 (PMC4043118; doi:10.1098/rsob.140044)
Supplement: Data refinement scheme [file rsob140044supp1.pdf]

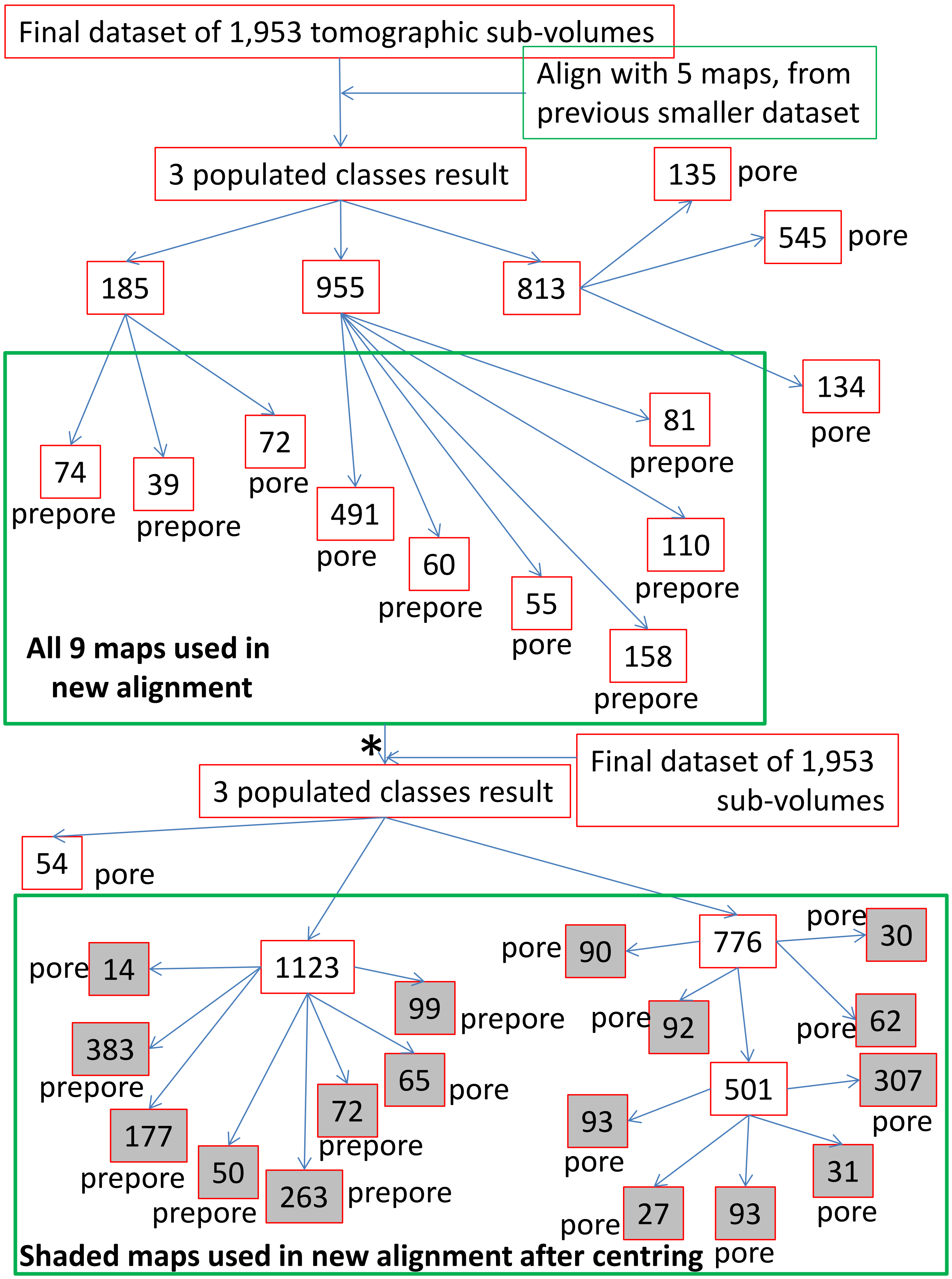
Final dataset of 1,953 tomographic sub-volumes
Align with 5 maps, from previous smaller dataset
3 populated classes result
185
955
813
135 pore
545 pore
134 pore
74 prepore
39 prepore
72 pore
491 pore
60 prepore
55 pore
81 prepore
110 prepore
158 prepore
All 9 maps used in new alignment
*
Final dataset of 1,953 sub-volumes
3 populated classes result
54 pore
1123
776
pore 14
383 prepore
177 prepore
50 prepore
263 prepore
72 prepore
65 pore
99 prepore
pore 90
pore 92
pore 30
pore 62
501
93 pore
27 pore
93 pore
31 pore
307 pore
Shaded maps used in new alignment after centring

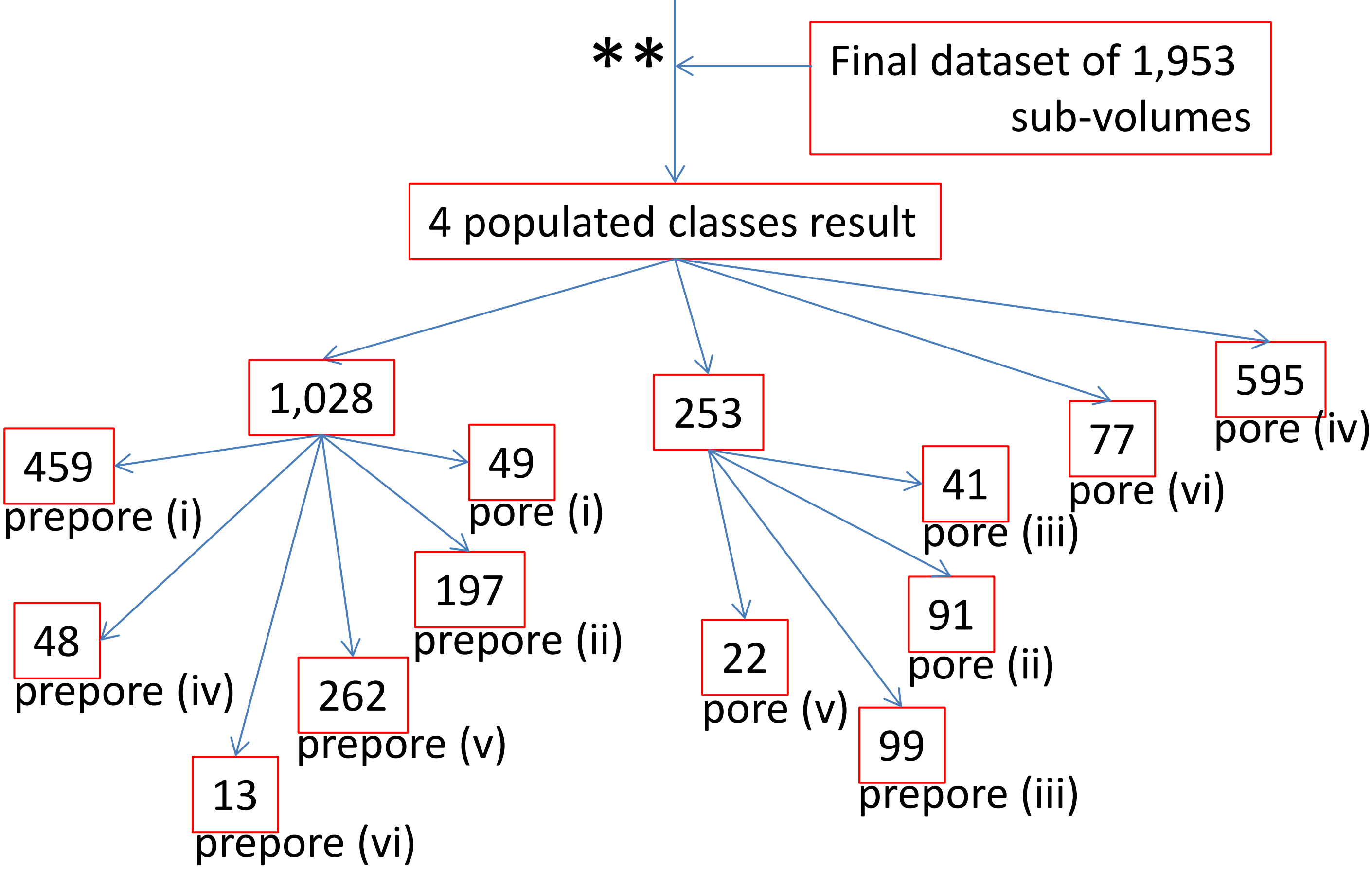
**
Final dataset of 1,953 sub-volumes
4 populated classes result
1,028
459
prepore (i)
49
pore (i)
197
prepore (ii)
48
prepore (iv)
262
prepore (v)
13
prepore (vi)
253
41
pore (iii)
91
pore (ii)
22
pore (v)
99
prepore (iii)
77
pore (vi)
595
pore (iv)
